# From Farm-to-Fork: *E. Coli* from an Intensive Pig Production System in South Africa Shows High Resistance to Critically Important Antibiotics for Human and Animal Use

**DOI:** 10.3390/antibiotics10020178

**Published:** 2021-02-10

**Authors:** Shima E. Abdalla, Akebe Luther King Abia, Daniel G. Amoako, Keith Perrett, Linda A. Bester, Sabiha Y. Essack

**Affiliations:** 1 Antimicrobial Research Unit, College of Health Sciences, University of KwaZulu-Natal, Durban 4000, South Africa; lutherkinga@yahoo.fr (A.L.K.A.); amoakodg@gmail.com (D.G.A.); essacks@ukzn.ac.za (S.Y.E.); 2 Biomedical Resource Unit, College of Health Sciences, University of KwaZulu-Natal, Durban 4000, South Africa; besterl@ukzn.ac.za; 3 Centre for Respiratory Diseases and Meningitis, National Institute for Communicable Diseases, Johannesburg 2131, South Africa; 4 Epidemiology Section, KwaZulu-Natal Agriculture & Rural Development-Veterinary Service, Pietermaritzburg 3201, South Africa; keith.perrett@kzndard.gov.za

**Keywords:** antibiotic susceptibility, *E. coli*, multidrug resistance, multiple-antibiotic resistance index, farm-to-fork, intensive pig farming, biosecurity, animal husbandry, antibiotic stewardship, South Africa

## Abstract

Antibiotic resistance profiles of *Escherichia coli* were investigated in an intensive pig production system in the uMgungundlovu District, South Africa, using the ‘farm-to-fork’ approach. Four hundred seventeen (417) samples were collected from pig and pig products at different points (farm, transport, and abattoir). *E. coli* was isolated and enumerated using the Colilert^®^ 18/Quanti-Tray^®^ 2000 system. Ten isolates from each Quanti-tray were selected randomly and putatively identified on eosin methylene blue agar. Real-time PCR targeting the *uid*A gene was used to confirm isolates to the genus level. The Kirby–Bauer disc diffusion method was used to determine the isolates’ antibiotic susceptibility profiles against 20 antibiotics. A total of 1044 confirmed *E. coli* isolates were obtained across the three critical points in the food chain. Resistance was observed to all the antibiotics tested with the highest and lowest rates obtained against tetracycline (88.5%) and meropenem (0.2%), respectively. Resistance was also observed to chloramphenicol (71.4%), ampicillin (71.1%), trimethoprim-sulfamethoxazole (61.3%), amoxicillin-clavulanate (43.8%), cephalexin (34.3%), azithromycin (23.9%), nalidixic acid (22.1%), cefoxitin (21.1%), ceftriaxone (18.9%), ciprofloxacin (17.3%), cefotaxime (16.9%), gentamicin (15.5%), cefepime (13.8%), ceftazidime (9.8%), amikacin (3.4%), piperacillin-tazobactam (1.2%), tigecycline (0.9%), and imipenem (0.3%). Multidrug resistance (MDR) was observed in 71.2% of the resistant isolates with an overall multiple antibiotic resistance (MAR) index of 0.25, indicating exposure to high antibiotic use environments at the farm level. A high percentage of resistance was observed to growth promoters and antibiotics approved for veterinary medicine in South Africa. Of concern was resistance to critically important antibiotics for animal and human use and the watch and reserve categories of antibiotics. This could have adverse animal and human health consequences from a food safety perspective, necessitating efficient antibiotic stewardship and guidelines to streamline antibiotic use in the food-animal production chain.

## 1. Introduction

Animal-based protein consumption continues to increase worldwide due to economic development and urbanization [1]. In 2013, the Food and Agriculture Organization (FAO) of the United Nations estimated the average annual consumption of meat per person was 48 kg, which translates to more than 50 billion animals to meet the world’s demand [2]. The global production of meat and meat products due to increasing demand is projected to increase by 76% by 2050 [3,4]. In developing countries, animal product consumption is expected to increase from 29% to 35% by 2030 and 37% by 2050 [5], leading to a substantial increase in livestock production [6]. The current meat production in developing countries remains insufficient to satisfy demand, particularly in Africa [7].

Meat demand increases antibiotics use as growth promoters, for prophylaxis and metaphylaxis in food animals [8], particularly in the absence of adequate biosecurity and animal husbandry programmes. Prophylaxis is the administration of antibiotics to healthy food animals to prevent diseases from occurring, while metaphylaxis is the treatment of clinically healthy pigs in the same group where some animals have shown clinical symptoms of the disease [9]. Antibiotic growth promoters (AGPs) are substances that kill or inhibit bacteria and are administered in subtherapeutic doses to enhance animals’ growth [10]. These AGPs are considered as the most cost-effective way of maintaining health and feed efficiency [11] as their use in livestock diets has increased the farmers’ and agricultural industries’ productivity [12] by decreasing the negative impacts of stress in livestock, such as post-weaning diarrhea in pigs [13].

However, the subtherapeutic use of antibiotics as growth promoters results in the development and subsequent transmission of resistant bacteria between animals and from animals to humans [14]. Many studies have shown that AGPs’ use affects the intestinal bacteria of the animals and leads to bacterial evolution, changes in bacterial survival, and bacterial gene mutations leading to antibiotic resistance development [15,16,17]. As a result, the European Union (EU) has restricted the use of AGPs since 1999 and completely banned their use in 2006 [18]. Both the World Organization for Animal Health (OIE) [19] and the World Health Organization (WHO) have compiled lists of critically important antibiotics for animal and human health to minimize the health risks associated with antimicrobial use and subsequent resistance in food-producing animals [20].

Intensive pig farming is considered a solution that may bridge the deficit in animal protein demand due to many factors such as rapid fecundity rates, high feed conversion, early maturity, short generation intervals, and small space requirements [21]. Pork has become an essential source of animal protein worldwide, and, within the last three decades, the consumption of pork increased by 70% in developing countries [22]. However, one disadvantage of farming the animals in a small space (typical of intensive farming) is the increased risk of infection transmission and stress in the animals, requiring antibiotics for treatment [23]. Thus, like many other food animals, pigs have become potential reservoirs for bacterial pathogens, including those that are resistant, even to clinically important antibiotics used in human medicine; these could be transferred to humans through pork or into the environment using porcine manure for agricultural purposes [24].

*Escherichia coli* is an enteric bacterium typically found in humans and animals’ gastrointestinal tracts, with a lifestyle ranging from commensal to obligate pathogen [25,26]. As a pathogen, it is associated with bacteremia, wound infections, urinary and gastrointestinal tract infections [27]. Both commensal and pathogenic *E. coli* have also been implicated in transferring resistance genes to other bacteria, including pathogenic ones [28]. Due to its presence in a wide range of hosts, *E. coli* is considered a useful indicator for the prevalence of antibiotic resistance, allowing the evaluation and comparison of the prevalence resistance between different populations and evaluating animal to human transmission [29]. Moreover, *E. coli* has been reported to serve as a reservoir of antibiotic resistance genes that could be transferred from animals to humans and provides vital information on the flow of resistance in the food chain [30,31].

Although the South African pork industry is not the largest in terms of the country’s overall food animal production sector [32], when combined with the poultry industry, these two sectors are considered as the highest consumers of antimicrobials for treatment, disease prevention, and growth-promotion [33]. In a previous study, we demonstrated that over 60% of *E. coli* isolated from an intensive poultry system was resistant to numerous antibiotics, including clinically relevant ones [23]. Nevertheless, this study was limited by the number of isolates that were tested. Additionally, compared to poultry, pigs have a more extended growth period that typically results in the administration of greater quantities of a broader range of antimicrobials [34]. Despite this, there is limited data on the antibiotic resistance in *E. coli* in intensive pig production in South Africa [35]. Therefore, it is essential to investigate the use of antimicrobials in such sectors and their impact on the emergence and escalation of resistance, as the transmission to humans could lead to infectious disease treatment failures. Using the ‘farm-to-fork’ approach, the current study aimed to investigate the antibiotic resistance profiles of *E. coli* isolated from an intensive pig production system in the uMgungundlovu District KwaZulu-Natal, South Africa. Most other pig studies focus on a single point along the continuum, such as the farm alone or slaughterhouse. However, the ‘farm-to-fork’ approach used in the current study gives a better picture of the distribution of resistance along the entire continuum, by allowing for samples to be collected from all points, beginning from the farm, through the transport to the final packaged product, usually involving the same batch of animals from the introduction into the farm to slaughter.

## 2. Results

### 2.1. Prevalence of E. coli along the Pig Production Chain

**Figure 1:**
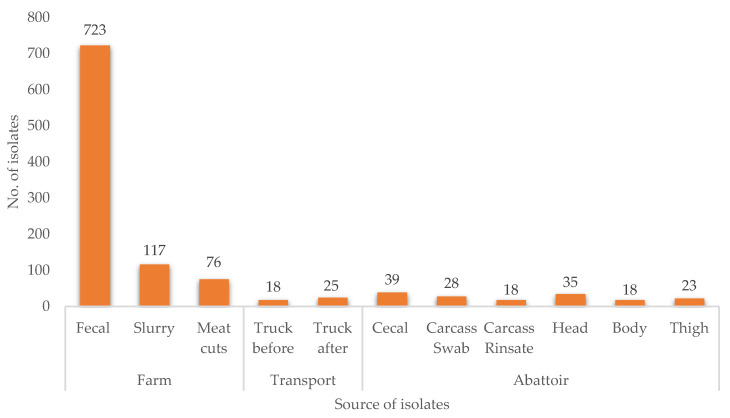
Distribution of *Escherichia coli* isolates along the pig production system, according to source and site.

*E. coli* was obtained at each critical point across the “farm to fork” continuum. The distribution of isolates along the continuum was 80.5% (*n* = 840), 4.1% (*n* = 43), and 15.4% (*n* = 161) for farm, transport, and abattoir, respectively (Figure 1).

### 2.2. Antimicrobial Resistance Profile of E. coli Isolated from the Pig Production Chain

**Figure 2:**
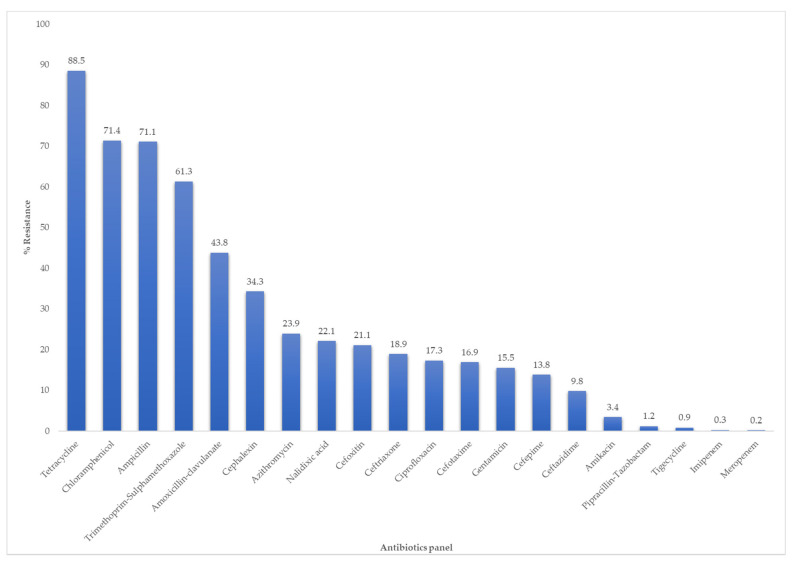
The overall percentage of antibiotic resistance in *E. coli* isolated across the pig production chain.

Ninety-eight percent (98.3%, *n* = 1027) of the isolates were resistant to at least one of the antibiotics tested. Resistance was observed to all the antibiotics and classes tested with the highest and lowest rate obtained against tetracycline (88.5%, *n* = 924) and meropenem (0.2%, *n* = 2), respectively (Figure 2).

**Figure 3:**
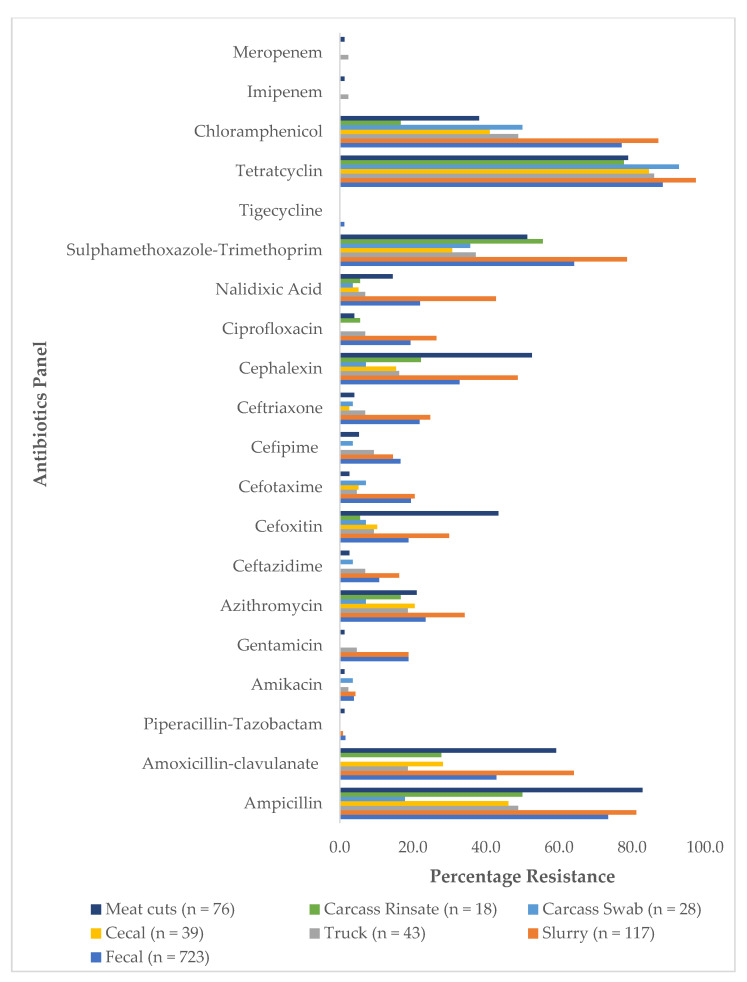
Percentage antibiotic resistance among isolates, stratified by source.

When stratified by site of collection, the highest percentage resistance was observed in the farm isolates. No tigecycline resistance was recorded in both truck and abattoir isolates (Figure 3).

The overall multidrug resistance (MDR) rate was 71.2% (*n* = 743). The highest prevalence of MDR was found on the farm 74.6% (*n* = 723) while truck and abattoir MDR rate was 51.1% (*n* = 43) and 50.3% (*n*= 161), respectively (Table 1). The difference in the MDR rate between the different sampling sites was statistically significant (Table 1). None of the isolates were pan-drug resistant (Table 1). Two hundred and two (202) MDR patterns were recorded in the study, the most common pattern being AMP-SXT-TET-CHL, found in 4.7% (*n* = 49) of the isolates (Table 1).

### 2.3. MAR Phenotypes of E. coli

**Figure 4:**
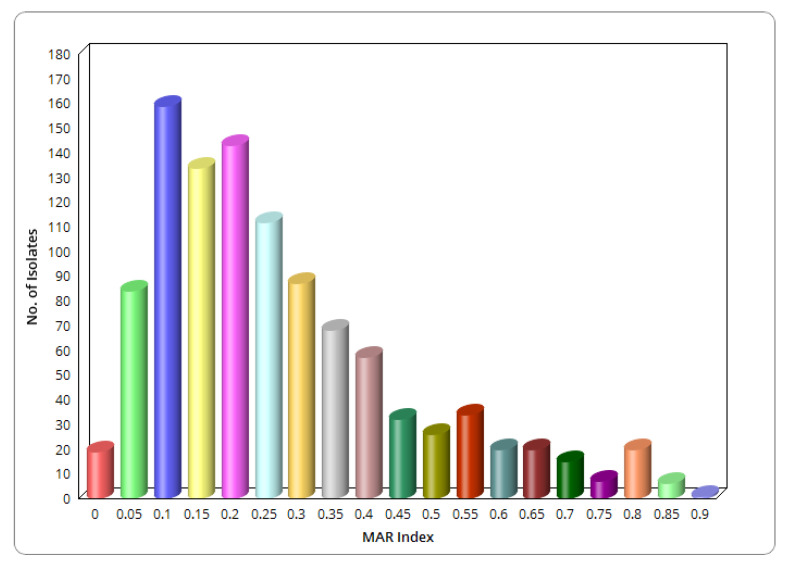
The multiple antibiotic resistance (MAR) indices of *E. coli* sampled across the farm-to-fork continuum.

The MAR indices ranged from 0.1 to 0.9. The lowest MAR index recorded among all the isolates was 0.1 (resistance to two antibiotics), and this was found in 15.2% (*n* = 157) of the total isolates. The highest MAR index was 0.9 (resistance to 18 antibiotics), recorded in 0.1% (*n* = 1) of the farm isolates. The highest MAR indices recorded in transport and abattoir were 0.7 and 0.75, respectively (Figure 4).

There was an overall statistically significant difference (*p* = 0.000; *p* < 0.05) between the MAR indexes from the different sampling (Table S1). The multiple pairwise comparison revealed statistically significant differences in the MAR indices between the farm and transport (*p* = 0.000; *p* < 0.05), and farm and abattoir (*p* = 0.004; *p* < 0.05). However, there was no statistically significant difference (*p* = 0.092; *p* < 0.05) between the transport and abattoir MAR indices (Table S1).

## 3. Discussion

We investigated the antibiotic resistance profiles of *E. coli* in an intensive pig production system in uMgungundlovu District, KwaZulu-Natal, South Africa using the farm-to-fork approach to determine the nature and extent of antibiotic resistance in food animal production. All the samples collected along the continuum were *E. coli* positive. The antibiotic sensitivity test revealed high resistance toward commonly used growth promoter analogues such as tetracycline, ampicillin, chloramphenicol, and sulfamethoxazole/trimethoprim. Of note, 76% of the *E. coli* isolates were multidrug-resistant (MDR). The MAR Index analysis intimated high exposure of isolates to antibiotics at the farm level.

### 3.1. Prevalence of E. coli across the Pig Production System

*Escherichia coli* was found across the farm-to-fork continuum with the highest number of positive samples observed on the farm. This was expected since farm samples constituted the largest proportion of the total sample, and the vast majority of samples were fecal. It was also unsurprising that *E. coli* was isolated from the transport vehicle as the pigs defecated during the transport. However, samples collected from the truck before the pigs’ transportation were also positive, indicating that the transport vehicle was not properly cleaned after the initial transport round. This could lead to the transfer of bacteria from one farm to another and the abattoir contributing to food contamination. It was not surprising to find *E. coli* in the cecal samples and the carcass rinsate at the abattoir. Contamination of carcasses by *E. coli* during the slaughter of animals leading to bacterial transmission through the food-chain has been reported [12,36,37,38]. The identification of *E. coli* in the meat samples in the meat processing area in the current study corroborates the findings of Schwaiger et al. [39]. They reported that almost 50% of the pork samples in their study were positive for *E. coli*, indicating that fecal contamination during the slaughter process could not be prevented entirely. Despite the careful removal of the animal’s internal organs, contamination from the intestinal contents would be challenging and unavoidable, likely due to many animals being processed. Therefore, stricter sanitary conditions should be observed in the abattoir to avoid transmitting these bacteria to consumers. The most appropriate approach would be to ensure proper rinsing of the carcass post-evisceration before sending them to the abattoir’s meat portion sections.

### 3.2. Antimicrobial Resistance Profile of E. coli Isolated from the Pig Production Chain

Although resistance to antibiotics is a natural phenomenon [34], their overuse and misuse in humans and animals have significantly escalated the antibiotic resistance levels [40]. In the current study, *E. coli* showed the highest resistance to tetracycline, chloramphenicol ampicillin, and sulfamethoxazole-trimethoprim, correlating with the use of amoxicillin sodium and trimethoprim-sulphamethoxazole reported by the farm (personal communication). Ampicillin, sulphonamides, and tetracycline have a long history of use in animals [41]. As mentioned by OIE Annual Report on Antimicrobial Agents Intended for Use in Animals (2019), the largest proportion of antibiotics used in animal production was tetracycline’s followed by ampicillin and macrolides [42]. In South Africa, tetracyclines are the most commonly used antibiotics in animals after the macrolides; they are registered as a growth promoter in the Fertilizers, Farm Feeds, Agricultural Remedies and Stock Remedies Act (Act 36 of 1947), providing a possible explanation for the resistance observed [43].

Although many studies conducted in other countries have reported similar percentage resistance in *E. coli* against the antibiotics tested, the resistance varies between countries based on antibiotics and the country’s regulations. For example, an Australian study [44] reported the highest resistance was toward tetracycline (68.2%), ampicillin (60.2%), chloramphenicol (47.8%), and trimethoprim/sulfamethoxazole (34.3%), respectively. However, these rates were relatively lower than in our study and reflected the strict use of antibiotics in Australian livestock production. Australia has been reported as one of the five lowest antibiotic users in livestock production globally [45]. On the contrary, a surveillance study in China investigating the antibiotic resistance trends in *E. coli* originating from food animals during 2008–2015 [41] reported a high resistance rate to tetracycline, sulfamethoxazole, and ampicillin at 94%, 88.36%, and 81.44%, respectively. This could be explained by the fact that in China, using antibiotics both for animal disease treatment and growth promotion is unmonitored [46].

The factors behind the emergence and spread of resistant bacteria are complex. They may be due to coselection, whereby using one antibiotic selects for resistance to other substances [47]. Such has been reported for chlortetracycline, penicillin, and sulfamethazine coselecting for antimicrobials of other classes that were not administered, like aminoglycoside [11]. Indeed, chloramphenicol is not registered for use in food animals in South Africa [48] but, high percentage resistance was still observed, possibly due to the horizontal transfer of chloramphenicol resistome and the coselection of resistance because of the use of other compounds [48,49]. Moreover, it has been found that resistance to sulfamethoxazole, tetracycline, and kanamycin is frequently transferred along with chloramphenicol resistance as the *cmA1* gene conferring resistance to chloramphenicol is cocarried with genes encoding resistance to other antimicrobials that are currently approved for use in food animals [50]. Although cephalosporins and quinolones showed a relatively low resistance rate in comparison to other antibiotics, they still need monitoring due to their clinical importance [51]. The WHO has classified both cephalosporins and quinolones as critically important antibiotics for human medicine [20].

Notwithstanding the low percentage resistance to carbapenems, the emergence of carbapenem resistance is grave as the WHO classifies them as critically important antibiotics [20]. Moreover, carbapenems are the last-resort antibiotics for treating a wide range of infections caused by multidrug-resistant Gram-negative bacteria [52].

### 3.3. Prevalence of Multidrug-Resistant and Estimation of MAR Index of E. coli Isolates in the Pig Production Chain

Food animal husbandry is considered an important factor contributing to the distribution of multidrug-resistant bacteria [53]. In this study, multidrug resistance was found in 71% of the total isolates. Almost 200 different antibiogram patterns were found along the continuum. The highest diversity in antibiogram patterns was found on the farm compared to both the transport and abattoir. The most prevalent MDR pattern reported mostly in the three different sampling areas included resistance to commonly used growth promoters and antibiotics in veterinary and human medicine belonging to the same class of antibiotics in different permutations and combinations, indicating the possibility of transmission along the pork production chain.

The development of resistance to these antibiotic classes is a major concern in human and animal medicine because these drugs are commonly used both in practices [54], especially as infections with MDR strains will limit treatment options [55]. The increasing prevalence of MDR *E. coli* is challenging because *E. coli* can occupy multiple niches, including humans and animals, thereby acquiring or transmitting antimicrobial resistance genes horizontally and vertically [56].

The multiple antibiotic resistance (MAR) index is used to determine the health risk associated with the spread of resistance in a specified location [54,57]. A MAR index of 0.2 differentiates between low- and high-risk, and a MAR index greater than 0.2 suggests that bacteria were exposed to high antibiotic use environments [58]. The mean MAR index of 0.29 on the farm affirmed the high antibiotic use and high selective pressure in the farm, which was statistically significant (*p*-value < 0.01) compared to the other sampling points. This could also indicate the possible transfer of the resistant bacteria along the production chain. Worryingly, results in this study revealed an increasing resistance to all antibiotic classes, including critically important antibiotics for human use and the watch and reserve categories of antibiotics leading to serious concern to human health [59,60]. Hence, this reiterates the calls for a holistic review on the use of these antibiotics and growth promoters in the food-animal production chain.

## 4. Materials and Methods

### 4.1. Study Clearance and Ethical Consideration

Ethical approval was received from the Animal Research Ethics Committee (Reference: AREC 073/016PD) and the Biomedical Research Ethics Committee (Reference: BCA444/16) of the University of KwaZulu-Natal. A section 20A permit was further obtained from the South African National Department of Agriculture, Forestry, and Fisheries (Reference: 12/11/1/5).

### 4.2. Study Site and Sample Collection

This longitudinal study was conducted over 18 weeks (September 2018 to January 2019) from birth to slaughter. The collection points consisted of the pig farm, transport system (truck), and the associated abattoir. A total of 417 samples were collected from these points following the World Health Organization Advisory Group on Integrated Surveillance of Antimicrobial Resistance (WHO-AGISAR) guidelines as follows:

- Farm: Two groups of newborn pigs in two fences labeled A and B were selected for the study. Five fresh pig feces samples were randomly collected per fence, ensuring that each sample was from a different location. Samples were collected twice a month for 18 weeks. Additionally, slurry samples were collected in triplicate from the pipes draining the pig house at each sampling period.
- Transport: After 18 weeks, when pigs reached maturity and slaughter readiness, swab samples were taken from the transport vehicle (truck) before and after loading the pigs for transportation to the abattoir.
- Abattoir: Swabs were collected throughout the slaughter chain, viz. carcass, carcass rinsate, caeca, and pork portions before packaging (head, body, and thigh).

All samples were transported on ice packs to the Antimicrobial Research Unit’s microbiology laboratory, University of KwaZulu-Natal, and processed within 4 h from the time of collection.

### 4.3. Sample Processing and Isolation of E. coli

*E. coli* was isolated using the Colilert^®^ 18/Quanti-Tray^®^ 2000 system (IDEXX Laboratories (Pty) Ltd., Johannesburg, South Africa) according to the manufacturer’s instructions.

#### 4.3.1. Fecal and Slurry Samples from the Farm

Each fecal sample (1 g) was weighed and transferred into 9 mL of distilled water and vortexed briefly. A total of 100 µL of the supernatant from the resuspended sample was transferred into a 120 mL sterile plastic bottle, and the bottle topped up to the 100 mL mark with sterile distilled water. The 100 mL sample was then processed as per the IDEXX protocol for water samples (IDEXX Laboratories (Pty) Ltd., Johannesburg, South Africa). For the slurry samples, 20 µL of the slurry were analyzed directly without any prior processing.

#### 4.3.2. Rinsate and Swabs (Transport and Abattoir)

**Figure 5:**
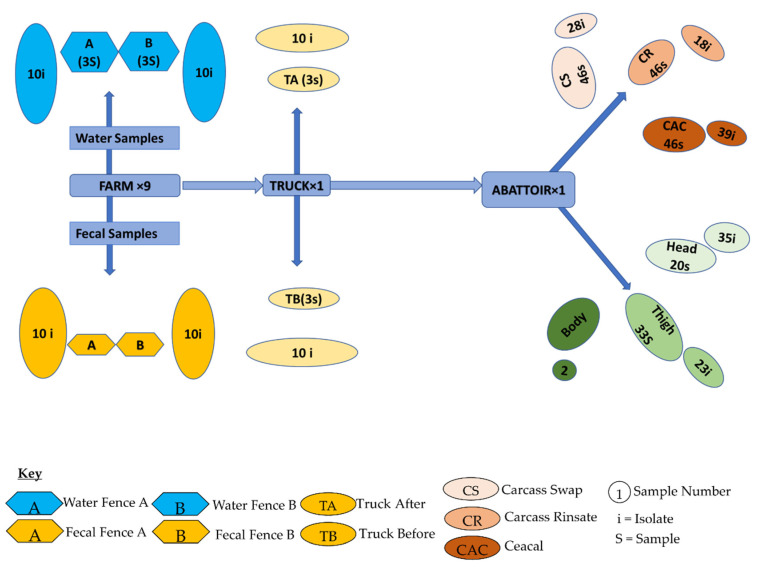
A flow diagram depicting the sampling frame used in the farm-to-fork approach.

For the carcass rinsate, 48 samples were pooled into four equal samples of 12 each, after which 1 mL dilutions were extracted and processed in the same way as the slurry samples. Carcass and meat cut swabs from each site were pooled into four sets of 12 and transferred into 10 mL sterile distilled water. The mixture was vortexed for 1 min to separate the bacteria from the swabs. The subsequent supernatant was then processed further as with the slurry samples. For the cecal samples, 48 different ceca samples were pooled into four samples of 12 each and mixed properly. Twenty-five grams of each mixture was transferred into a sterile container containing 225 mL of sterile distilled water. The mixture was vigorously shaken manually, and then 20 µL of the supernatant was extracted and processed like the slurry samples. A flow diagram depicting the sampling frame used in the farm-to-fork approach is shown in Figure 5.

### 4.4. Molecular Confirmation of E. coli

All the samples collected along the continuum were positive for *E. coli*, using the Colilert^®^ 18/Quanti-Tray^®^ (data not shown) as mentioned previously. Ten isolates from each Quanti-tray were selected randomly and putatively identified on eosin methylene blue agar for further confirmation by PCR, yielding a final sample size of 1044 *E. coli* isolates. DNA was extracted from these isolates using the boiling method [61]. The extracted DNA was used as a template to confirm *E. coli* using real-time polymerase chain reaction (PCR), targeting the *uidA* gene. The reactions were performed in a total volume of 10 µL consisting of 5 µL of Luna^®^ universal qPCR master mix (New England Biolabs, Ipswich, MA, USA), 0.5 µL from each primer (forward-AAAACGGCAAGAAAAAGCAG and reverse-ACGCGTGGTTAACAGTCTTGCG; final concentration 0.5 µM (Inqaba Biotechnical Industries (Pty) Ltd., Pretoria, South Africa)), 3 µL DNA, and 1 µL of nuclease-free water. The thermal cycling conditions were as previously described [62]. After the final extension step, a melt curve was generated and analyzed as previously described [63]. All reactions were performed on a Quant Studio^®^ 5 Real-time PCR system (Thermo Fischer Scientific, Waltham, MA, USA). DNA from *E. coli* ATCC^®^ 25922 was used as a positive control, while the reaction mixture with no DNA (replaced with nuclease-free water) was used as a no template control.

### 4.5. Antibiotic Susceptibility Testing

Antibiotic susceptibility profiles were determined using the Kirby–Bauer disk diffusion method on Muller Hinton Agar (Oxoid, Basingstoke, Hampshire, England). Twenty antibiotics were tested and included the β-lactams made up of penicillins (ampicillin (AMP 10 µg)), penicillin-inhibitor combinations (amoxicillin-clavulanate (AMC 20/10 µg), piperacillin-tazobactam (TZP 30/6 µg)), cephalosporins (cephalexin (LEX 30 µg), ceftriaxone(CRO 5 µg), cefotaxime (CTX 10 µg), cefepime (FEP 30 µg), ceftazidime (CAZ 10 µg)), cephamycins, (cefoxitin (FOX 10 µg)), carbapenems (meropenem (MEM 10 µg), and imipenem (IMP 10 µg)), macrolides (azithromycin (AZM 15 µg)), aminoglycosides (gentamicin (GEN 10 µg), amikacin (AMK 30 µg)), tetracycline (tetracycline (TET 30µg), tigecycline (TGC 15 µg)), amphenicols (chloramphenicol (CHL 30 µg)), sulphonamides (trimethoprim-sulfamethoxazole (SXT 1.25/23.7µg)), and quinolones (ciprofloxacin (CIP 5 µg), nalidixic acid (NAL 30 µg)). *E. coli* ATCC^®^ 25922 was used for quality control. The diameters of the inhibition zones were measured and interpreted according to the European Committee on Antimicrobial Susceptibility testing breakpoints except for azithromycin, nalidixic acid, and tetracycline where the Clinical and Laboratory Standards Institute guidelines were used. All the antibiotic discs were purchased from Oxoid (Basingstoke, Hampshire, England).

### 4.6. Determination of Multidrug-Resistant (MDR) and Multiple Antibiotic Resistance (MAR) Index

Isolates showing resistance to ≥1 agent in >3 antibiotic classes were considered as multidrug-resistant (MDR) [64]. The Multiple Antibiotic Resistance (MAR) index was calculated as a/b, where ‘a’ was the number of antibiotics to which an isolate was resistant, and ‘b’ was the total number of antibiotics tested [65].

### 4.7. Statistical Analysis and Interpretation

The data were analyzed using the Statistical Package for the Social Science SPSSv26 (IBM, Armonk, NY, USA). Descriptive statistics were used to describe the frequency of *E. coli* that was isolated from different sources. The prevalence of MDR isolates and MAR index of *E. coli* from different sampling sources were compared using the ANOVA, Tukey test, and a *p*-value of < 0.05 was considered statistically significant.

## 5. Conclusions

To our knowledge, this is the first study in South Africa investigating antibiotic-resistant *E. coli* in intensive pig farming using the “farm-to-fork” approach. *E. coli* showed high percentage and multidrug resistance with high MAR indices, suggesting that the isolates originated from high antibiotic use/exposure areas. Of note, high percentage resistance was observed to growth promoters and antibiotics approved for use in veterinary medicine in South Africa. This could have adverse human health consequences from a food safety perspective, necessitating efficient antibiotic stewardship and guidelines to streamline antibiotic use. Therefore, it is recommended that strict regulations regarding the use of antibiotics in food animals in South Africa be developed and implemented to curb this issue.

## Figures and Tables

**Table 1:** Table depicting the most frequent multidrug-resistant (MDR) profiles of *E. coli.*

Antibiograms	Frequency
AMP-SXT-TET-CHL	49
AMP-TET-CHL	44
AMP-AMC-TET-CHL	31
AMP-AMC-SXT-TET-CHL	27
AZM-SXT-TET-CHL	27
SXT-TET-CHL	22
AMP-AMC-AMK-GEN-AZM-CAZ-FOX-CTX-FEP-CRO-LEX-CIP-NAL-SXT-TET-CHL	18
AMP-AMC-GEN-CIP-NAL-SXT-TET-CHL	16
AMP-SXT-TET	16
AMP-AMC-LEX-SXT-TET-CHL	13
AMP-AMC-LEX-TET-CHL	13
AMP-GEN-CIP-NAL-SXT-TET-CHL	11
AMP-AMC-AZM-CL-SXT-TET-CHL	10
AMP-AMC-GEN-CAZ-FOX-CTX-FEP-CRO-LEX-CIP-NAL-SXT-TET-CHL	10
AMP-AZM-LEX-SXT-TET-CHL	10
AZM-TET-CHL	10

## Supplementary Materials

The following are available online at https://www.mdpi.com/2079-6382/10/2/178/s1, Table S1: MAR index and MDR of the *E. coli* isolates across the food chain.
