# Supplementary material for: From Farm-to-Fork: E. Coli from an Intensive Pig Production System in South Africa Shows High Resistance to Critically Important Antibiotics for Human and Animal Use
Source: Antibiotics (Basel). 2021 Feb 10;10(2):178. doi: 10.3390/antibiotics10020178 (PMC7916376; doi:10.3390/antibiotics10020178)
Supplement: Supplementary file 1 [file antibiotics-10-00178-s001.pdf]

antibiotics

MDPI

From farm-to-fork: *E. coli* from an intensive pig production system in South Africa shows high resistance to critically important antibiotics for human and animal use

Shima E. Abdalla1*, Akebe Luther King Abia1, Daniel G. Amoako1, 2, Keith Perrett3, Linda A. Bester2 and Sabiha Y. Essack1

[1] Antimicrobial Research Unit, College of Health Sciences, University of KwaZulu-Natal, Durban 4000, South Africa; shimaeltayeb23@gmail.com (S.E.A.); lutherkinga@yahoo.fr (A.L.K.A.); amoakodg@gmail.com (D.G.A.); essacks@ukzn.ac.za (S.Y.E.).
[2] Biomedical Resource Unit, College of Health Sciences, University of KwaZulu-Natal, Durban 4000, South Africa; besterl@ukzn.ac.za (L.A.B.)
[3] Epidemiology Section, KwaZulu-Natal Agriculture & Rural Development-Veterinary Service; Pietermaritzburg 3201, South Africa; keith.perrett@kzndard.gov.za (K.P.)
* Correspondence: shimaeltayeb23@gmail.com (S.E.A.)

Table S1: MAR index and MDR of the *E. coli* isolates across the food-chain.

Source	Number of isolates	MAR index		Overall ANOVA *p*-value	Pairwise Tukey HSB comparison		
		Mean	Std. Deviation		Farm vs Transport	Farm vs Abattoir	Transport vs Abattoir
Farm	840	0.2888	0.19415	0.000***	0.000***	0.004**	0.902
Transport	43	0.1686	0.16584				
Abattoir	161	0.1823	0.12455				
Host							
Pig (n=1044)							
Mean= [0.25]							
Other Parameters							
Mode= [0.10]							
Median= [0.25]							
MDR= 76.10%							
Pan-susceptible= 1.70%							
≥1 Antimicrobial= 98.30%							
≥5 Antimicrobial= 48.40%							
≥10 Antimicrobial= 14.30%							
≥15 Antimicrobial= 3.30%							
Pan-resistance= 0.00%							

Keys: Statistical significance: (*) *P*-value <0.05; (**) *P*-value <0.01; (***) *P*-value <0.001
